# Familiarity Breeds Contempt: Kangaroos Persistently Avoid Areas with Experimentally Deployed Dingo Scents

**DOI:** 10.1371/journal.pone.0010403

**Published:** 2010-05-05

**Authors:** Michael H. Parsons, Daniel T. Blumstein

**Affiliations:** 1 Centre for Ecosystem Diversity and Dynamics (CEDD), Department of Environmental Biology, Curtin University, Perth, Western Australia, Australia; 2 School of Veterinary Biology and Biomedical Sciences, Murdoch University, Murdoch, Western Australia, Australia; 3 Department of Ecology and Evolutionary Biology, University of California Los Angeles, Los Angeles, California, United States of America; University of Utah, United States of America

## Abstract

**Background:**

Whether or not animals habituate to repeated exposure to predator scents may depend upon whether there are predators associated with the cues. Understanding the contexts of habituation is theoretically important and has profound implication for the application of predator-based herbivore deterrents. We repeatedly exposed a mixed mob of macropod marsupials to olfactory scents (urine, feces) from a sympatric predator (*Canis lupus dingo*), along with a control (water). If these predator cues were alarming, we expected that over time, some red kangaroos (*Macropus rufous*), western grey kangaroos (*Macropus fuliginosus*) and agile wallabies (*Macropus agilis*) would elect to not participate in cafeteria trials because the scents provided information about the riskiness of the area.

**Methodology/Principal Findings:**

We evaluated the effects of urine and feces independently and expected that urine would elicit a stronger reaction because it contains a broader class of infochemicals (pheromones, kairomones). Finally, we scored non-invasive indicators (flight and alarm stomps) to determine whether fear or altered palatability was responsible for the response. Repeated exposure reduced macropodid foraging on food associated with 40 ml of dingo urine, *X* = 986.75±3.97 g food remained as compared to the tap water control, *X* = 209.0±107.0 g (*P*<0.001). Macropodids fled more when encountering a urine treatment, *X* = 4.50±2.08 flights, as compared to the control, *X* = 0 flights (*P*<0.001). There was no difference in effect between urine or feces treatments (*P*>0.5). Macropodids did not habituate to repeated exposure to predator scents, rather they avoided the entire experimental area after 10 days of trials (*R* ^2^ = 83.8; *P*<0.001).

**Conclusions/Significance:**

Responses to urine and feces were indistinguishable; both elicited fear-based responses and deterred foraging. Despite repeated exposure to predator-related cues in the absence of a predator, macropodids persistently avoided an area of highly palatable food. Area avoidance is consistent with that observed from other species following repeated anti-predator conditioning, However, this is the first time this response has been experimentally observed among medium or large vertebrates − where a local response is observed spatially and an area effect is revealed over time.

## Introduction

Many animals assess risk from intra-specific scent cues left behind by potential predators. Sulfur containing chemicals, volatile fatty acids and ketones (all diet released metabolites) may cause the repellent properties of predator urine and feces [1]. However, urine and anal scent gland exudates also contain a broad class of infochemicals [2], including steroid alcohols and carrier proteins, that may synergistically indicate the: reproductive status [3], territorial status [4], age [5] social and nutritional status [6], and a time-stamp of an animal's presence (time since void/excretion) [7]. These complex properties likely evolved to assist intra-specific communication without alerting potential prey to the predator's presence. However, heterospecific eavesdropping, where potential prey species respond to such predator-secreted olfactory cues, has been demonstrated in invertebrates [8], fish [9], amphibians [10], birds [11], and mammals [12]. Animals can also discriminate urine from closely related species. For instance, foraging beavers (*Castor fiber*) respond to odors from the wolf (*Canis lupus*), but not dogs (*Canis familiaris*; [13]). Similarly, western grey kangaroos (*Macropus fuliginosis*) can discriminate between urine from a coyote (*Canis latrans*) and dingo (*Canis lupus dingo*; [14]). However, less is known about the chemical composition and stability of the messages contained within predator wastes, and this knowledge gap makes assessing the use of chemical cues as foraging deterrents difficult. For instance, there are extensive debates on the mechanism for deterrence [15], the time to habituation [16], and the likelihood of using this technology to train orphaned or predator naïve animals to avoid predators when re-introduced into the wild [17].

Prey may habituate (decline in responsiveness over repeated exposure) to the presence of the cue when not accompanied by the predator. For instance, bank voles (*Clethrionomys glareolus*) avoided areas with least weasel (*Mustela nivalis*) scent the most on the first day of a multi-day trail [18]. Goats (*Capra hircus*) habituated to tiger (*Panthera tigris*) feces as early as the third trial following repeated exposure [19]. Cape ground squirrels (*Xerus inauris*) quickly habituate to odors from black backed jackals (*Canis mesomelas*; [16] while mountain beavers (*Aplodontia rufa*) rapidly habituate to synthetic predator scents [20]. Even invertebrates have the ability to habituate; isopods habituate to sunfish (*Lepomis megalottis*) chemicals in 3 days [21].

Nonetheless, the presence of predator scents may influence patch selection [22], particularly when critical resources aren't being guarded [23]. Vilhunen [24] found that Arctic charr (*Salvelinus alpinus*) exposed only four times to cues from predatory pikeperch (*Sander lucioperca*) increased their spatial avoidance to pikeperch. Mongolian gerbils (*Meriones unguiculatus*; [25]), bank voles (*C. glareolus*;[26]), European hedgehog (*Erinaceus europae*; [26]), and house mice (*Mus domesticus*; [27]), avoid cue-laden habitats following repeated exposure to predatory cues. Few studies have demonstrated that medium -sized or large mammals have abandoned cafeteria trial areas. Moose (*Alces alces*) abandoned more than 50% of experimental areas following repeated exposure to urine from wolf (*Canis lupus*) and grizzly bears (*Ursus arctos horribilis*; [28]) and goats avoided ‘landscapes of fear’ laced with caracal dung (*Felis caracal*) however, indirect vulnerability cues (habitat features) were associated with the response [29].

It is essential to differentiate between habituation to chemical cues and the loss of potency of an aging cue; both could lead to the observation of decreased responsiveness. Highly volatile and less volatile agents combine to form complex scents [30]. For example, Brown hyenas (*Hyaena brunnei*) paste two different scents on the same blade of grass, one dissipates within two days and one lasts a month [31]. Carnivore scents are refreshed regularly in the wild [4] and it is likely that volatiles which advertise a time component of the scent are rapidly lost [32]. If the olfactory secretion contains differentially volatile compounds, the secretion may function as a “time-stamp” [7].

Different methods to study the response to olfactory secretions have unique constraints. Giving up densities (GUD; [33]) do not provide insight into the actual mechanism for deterrence because it does not demonstrate whether fear is responsible for the deterrent effect, only that animals stopped foraging at a particular time. For instance, sheep (*Ovis aries*) avoid food near domestic dog feces (*Canis familaris*; [34]; [35]). However, an aversive response was also observed from pig feces (*Sus domesticus*) during the same trials. The study concluded that wolf and dog feces were more heavily avoided than feces from non-predators. However carnivore feces are generally more pungent than herbivore feces, and thus dog feces may have simply been more volatile and noxious resulting in a higher level of reduced palatability. Kimball and Nolte [36] noted the common use of feces placed alongside experimental food troughs in GUD experiments, and have suggested that altered palatability is often misinterpreted as fear. And, when rabbits demonstrated a GUD response to fox, but not sheep feces, a second measure using fecal cortisol levels was necessary to characterize the response [37]. Similarly, Pyare and Berger [28] have used three escalating levels to characterize moose (*Alces alces*) response to Grizzly bear (*Ursus arctos horribilis*) urine; vigilance, pilo-erection, and avoidance or site abandonment were all necessary to describe the complete response. Among macropodids, flight from an area [38] may be the most apparent (and useful) measure of response to threat.

Different cues from the same predators may elicit dissimilar responses. For instance, fecal odors may generate different responses than those elicited by urine and dander. Both urine and feces from wolves (*Canis lupus*) and African lions (*Panthera leo*) produce repellent effects, possibly due to sulfurous meat metabolites in both substances [34]. However, Masini et al. [39] has shown that rats were able to discriminate among fur, urine and feces from ferrets (*Mustela nigripes*); fur created the strongest defensive response. Strangely, the repellent effects of hair and dander [1] are not related to the activity of sulphurous chemicals. Sullivan [40] has shown that snowshoe hares, (*Lepus americanus*) show a highly aversive response to wolverine (*Gulo gulo*) urine, demonstrate no response to feces, and only a moderate response to anal gland scents.

We previously observed western grey kangaroos (*Macropus fuliginosus*) to be deterred from food sources using urine from a sympatric predator (dingo, *Canis lupus dingo*); we were unable to elicit similar responses from coyote (*Canis latrans*) or human urine [14]. The aim of this study was to characterize the observed effects (fear or noxious-based avoidance), to understand the effects of repeated exposure over time, and to determine whether kangaroos can discriminate between different predator cues from the same species.

## Results

**Figure 1:**
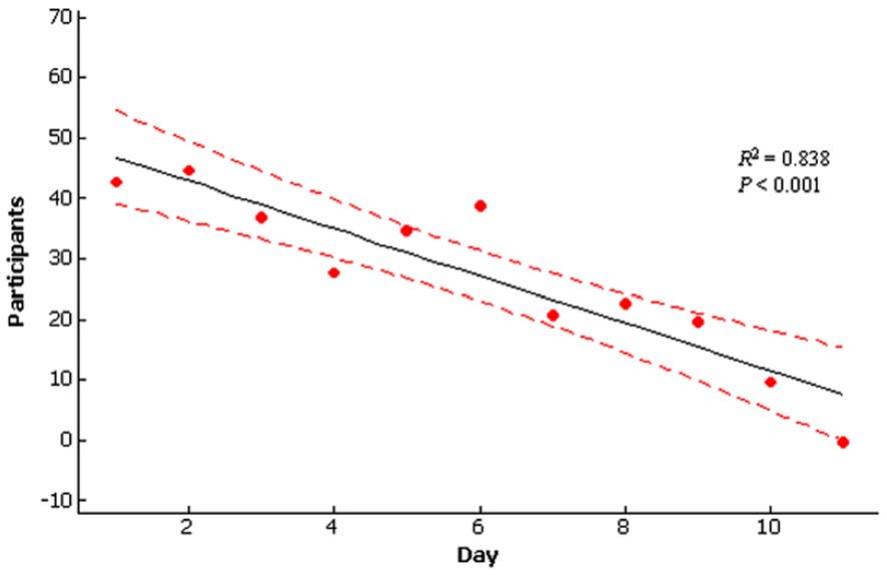
Participation over time. Participation by Western grey kangaroos (*Macropus fuliginosus*) Red kangaroos, (*Macropus rufous*) and Agile wallabies (*Macropus agilis*) attracted to trial area over a period of 11 days.

Kangaroo participation dropped steadily throughout the trial period (*R* ^2^ = 83.8; F_1,9_ = 46.46; *P*<0.001; Figure 1). A maximum of 45 individuals participated during the first day, and by day 11, no kangaroos elected to participate in the experiment (*X* = 27.36±4.27 individuals). There were significant increases in all between – subject effects following treatments (Table 1); MANOVA_(flight)_: *F*_2,3_ = 483.55, *P*<0.001; MANOVA_(alarm)_: F_2,3_ = 146.98, *P*<0.001; MANOVA_(encroach)_: F_2,3_ = 13.966, *P* = 0.006; and in the level of food remaining MANOVA_(GUD)_: F_2,3_ = 55.25, *P*<0.001. Participation did not vary by specific treatment: F_2,3_ = 6.41, *P*>0.5, but rather, macropodids reduced their overall participation over time.

**Table 1:** MANOVA for behavioral responses and GUD (biomass remaining) following presentation of two scent cues from the same predator.

	Response	Sum of Squares	df	*F*	*P*
MANOVA	Overall		6	22.533	0.001
	Flight	28.686	2	483.551	<0.001
Treatment	Stomps	26.544	2	144.981	<0.001
	Encroach	27.110	2	13.966	0.006
	Biomass (g)	8.482	2	55.245	<0.001
Participation	Flight	0.060	1	2.031	0.204
	Stomps	0.084	1	0.918	0.375
	Encroach	0.189	1	0.195	0.674
	Biomass (g)	0.001	1	0.007	0.937
Participation * Treatment		12.81	2	0.038	0.963

**Figure 2:**
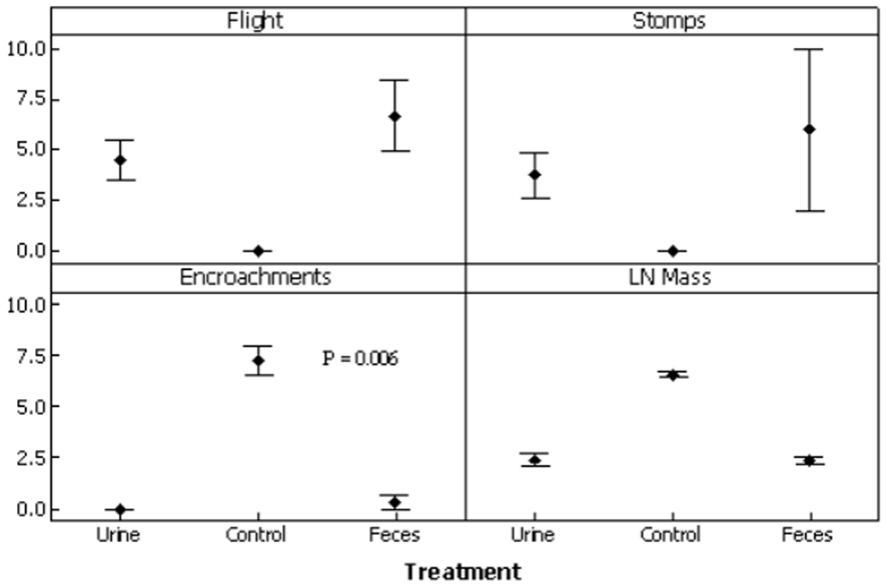
Indicators of fear. Effects of three behavioral measures (X ± SEM) to quantify vigilance as an indicator of fear; *P*<0.001 for all responses except as indicated. Y-axis is average frequency of instances for each indicator: flight, stomps, feeds from treatment trough, or LN mass removed (g).

Urine and feces generated strong effects as compared to the control for each response, though they did not differ from one another. Flight: Macropodids fled more when encountering a urine treatment, *X* = 4.50±2.08 flights, compared to the control *X* = 0 flights (*P*<0.001; Table 2, Figure 2). There were no detectable flight differences when encountering a fecal treatment, *X* = 6.67±3.055 flights (*P* = 0.444); the control was different to urine (*P*<0.001) and to feces (*P*<0.001). Alarm Stomps: Macropodids generated more alarm stomps when encountering a urine treatment, *X* = 3.75±2.21 stomps, as compared to the control *X* = 0 stomps (*P*<0.001). There were no detectable flight differences when encountering a fecal treatment, *X* = 6.00±6.92 stomps (*P* = 1.0); the control was different to both urine (*P*<0.001) and feces (*P*<0.001). Encroachment: Macropodids encroached less when encountering a urine treatment, *X* = 0 than a control, *X* = 9.00±0.676 encroaches (*P* = 0.002), but there was no difference between feces *X* = 0.33±0.33 and urine (*P* = 0.385); the control was different to both urine (*P*<0.001) and feces (*P*<0.001). GUD: Macropodids removed less food from the trough beside the urine treatment, *X* = 986.75±3.97 g as compared to the control trough, *X* = 209.0±107.0 g (*P*<0.001), but there was no difference between urine and feces, *X* = 988.67±2.03 g treatments (*P* = 1.0). The effect size of comparisons (*d*-scores) for each response was large between the urine and control, and between the feces and control, and negligible between urine and feces (Table 3).

**Table 2:** Pairwise comparisons (Tukey's HSD) for behavioral responses and GUD (biomass remaining) following presentation of two scent cues from the same predator.

Tukey's HSD	Contrast pairs		*P*
Flight	Urine	control	<0.001
	Urine	feces	0.444
	Feces	control	<0.001
Alarm Stomps	Urine	control	<0.001
	Urine	feces	0.966
	Feces	control	<0.001
Encroachment	Urine	control	0.002
	Urine	feces	0.385
	Feces	control	0.014
Biomass (g)	Urine	control	<0.001
	Urine	feces	1.000
	Feces	control	<0.001

**Table 3:** Effect size (d-scores) comparing the response of kangaroos following presentation of two scent cues (urine, feces) from the same predator.

Dependent variable	Urine- control	Feces- control	Urine- feces
Flight	2.79	4.00	2.41
Stomps	2.18	1.33	−0.75
Encroachments	−206	−15.0	−1.11
GUD	6.64	5.93	0.13

## Discussion

We found that visitation to the experimental array dropped steadily throughout the trial despite the attractive foods on offer, and despite the close proximity between treatment and control troughs. Indirect cues (shelter, resources, and lack of direct experience with predators) [29] may counter the intrinsic aversion – for a time. An area effect may not become apparent until repeated exposures reinforce memory. We are unaware of any other instances where area-affects were illuminated through temporal, rather than strictly spatial, responses. This finding is in strong contrast to expectations that animals rapidly habituate to predator cues [18]; [16]; [20]. We did not observe any habituation during these trials. This strengthens our concerns that subtle changes in chemical integrity over time [7], [30] may be falsely interpreted as habituation. We previously established that novel odors such as human and coyote (*Canis latrans*) urine did not produce alarm responses from western grey kangaroos, thus subjects learned to avoid an area that contained more threatening stimuli [24], possibly because alternative natural resources were available *ad libitum* in other park areas [23].

Area avoidance is consistent with that observed following repeated anti-predator conditioning of Arctic charr (*Salvelinus alpinus*) [24]. Space use was also modified with bank voles (*Clethrionomys glareolus*) [26] and spiny mice (*Acomys spp*.) [41] in the presence of predator odors. To our knowledge, however, area avoidance (third order responses; Table 4) from predator cues has rarely been experimentally demonstrated among medium or large sized mammals exposed to predator cues in cafeteria trials.

**Table 4:** Summary of cafeteria experiment to evaluate fear and avoidance responses among kangaroos to different dingo waste cues.

		Inferred state	Treatments	Covariate
Variable	Flight	Vigilance/fear	Dingo urine	Participation
	Alarm Stomp	Vigilance/fear	Dingo feces	
	Encroachment	Attraction	Tap water	
	Biomass removed	Attraction		
		**Implication**	**Example of behavior**	
Response level	First order	Awareness	Ignore food	
	Second order	Discrimination	Flight	
	Third order	Avoidance	Site abandonment	

Macropodids fed substantially less when a predatory cue was present compared to the water control. As has previously been observed [14], 68–90% of the food was taken from the trough during presentation of the tap water control, while no food was removed from the treatment troughs. Our indicators of fear, flight and alarm-stomps, were observed during all treatment periods and absent during control periods. This is not the first time that animals emitted alarm signals following presentation of an odor cue; meerkats (*Suricata suricata*) produce alarm calls following exposure to predator odor [42]. We wonder whether the alarm stomps acted synergistically in concert with the olfactory smell to increase the area of effect for congeners and conspecifics. For instance, when crayfish are exposed to a predator odour and alarm signal simultaneously, effects are increased [43]. We are confident that we have demonstrated that fear, rather than altered palatability [36], was responsible for these results.

We detected no difference in the response to dingo urine and feces; both were evocative. By constrast, dingo feces failed to produce an effect on the feeding rate of red-necked wallabies (*Macropus rufogriseus*) in an enclosure [17]. The structural integrity of the chemical cue (either frozen or fresh; [44] may have been responsible for the disparity between these different studies. We also note that some, but not all, canine feces are treated with exudates from the anal scent glands [4], thus a higher level of infochemicals may have been present in either sample.

The close proximity by which captive macropodids initially approached treatments was unexpected. Urine constituents (including pheromones) may be perceived up to 1 km from the source [7], and the olfactory capabilities of red kangaroos (*M. rufous*) have been compared to ‘the sharks’ ability to detect a drop of blood in water' [45]. Yet, we observed animals investigating to within 20 cm before reacting to the source cue. Animals may have been drawn to the scent by volatiles and then examined non volatiles for further information. We are also curious as to why some animals demonstrate vigilance (ears erect, pentapedal gait) following the investigation of the source cue, but still consume food at untreated troughs a few meters away. These findings confound the traditional (spatial) notion of ‘area effect’.

To maximize the value of fear based cues in rehabilitation and training contexts, further research is required to better understand: temporal and spatial interactions, cumulative effects of fear cues and microhabitat features (indirect cues), mechanisms to habituation, and the chemical stability of the signal. Due to species- specific responses to predator cues and the rarity of some natural predators, it may be necessary to artificially recreate (synthesize) active chemicals in a way that approximates the natural signal and context of application. A comparison of overlapping constituents in urine and feces (that have been treated with anal scent gland exudates) may assist identification of these compounds. Ultimately, artificial predator cues may be most useful in acute applications and are not intended to replace the ecological value of apex predators.

## Materials and Methods

Trials were carried out at the Caversham Wildlife Park (CWP; 31°85′39.5″ S, 115°97′45.1″ E), a commercial wildlife park 18 km N of Perth, Australia. CWP is located within a 3,600 ha conservation and leisure reserve. Seventy–two macropodid marsupials; 50 Red kangaroos (*Macropus rufous*) 20 western grey kangaroos (*Macropus fuliginisus*) and 2 agile wallabies (*Macropus agilis*) inhabited a 9 ha area. There were no apparent or detectable patterns in distribution except when macropodids congregated each morning to feed in the public lawn prior to public visitation. Among the red kangaroos, there were 30 females and 20 males, ages ranged from 4 months out of the pouch to 20 yrs. There were 10 males and 10 female adult western greys whose ages ranged from 2 to 20 yrs. There was one male and one female agile wallaby; both were <2 yrs. old. The macropodids had free access to water, herbage and shrubs and all were considered healthy. Experiments were conducted in compliance with the National Health and Medical Research Council (NHMRC) of Australia's code of practice for protecting animal welfare during research and followed ASAB/ABS guidelines for use. The Animal Ethics permit was granted by Curtin University of Technology; AEC R68-06.

Dingo urine and feces were collected from the Australian Dingo Conservation Association (ADCA) in Michelago, NSW. Animals spent evenings housed in a concrete lined shelter where pooled evening voids drain into a central repository (1 L Schott bottles). All urine and feces were collected fresh in 1 L Schott bottles and stored at 2°C. Fecal samples are commonly frozen for predator based cafeteria trials. However, we chose not to freeze samples due to the possibility of denaturing urine ‘carrier’ proteins during the freezing process [44]. To control for the loss of signal activity over time [7]; [32], we staggered our order of urine and feces (fresh urine and feces arrived weekly) to keep all treatments less than six-weeks old from time of collection. We have previously been unable to elicit fearful responses (flight, alarm stomps) using domestic dog urine (unpublished data) thus, for this study, we only used samples from pure bred dingoes [46]. Animals were fed a standard diet of chicken carcasses prior to sample collection.

### Feeding trials

**Figure 3:**
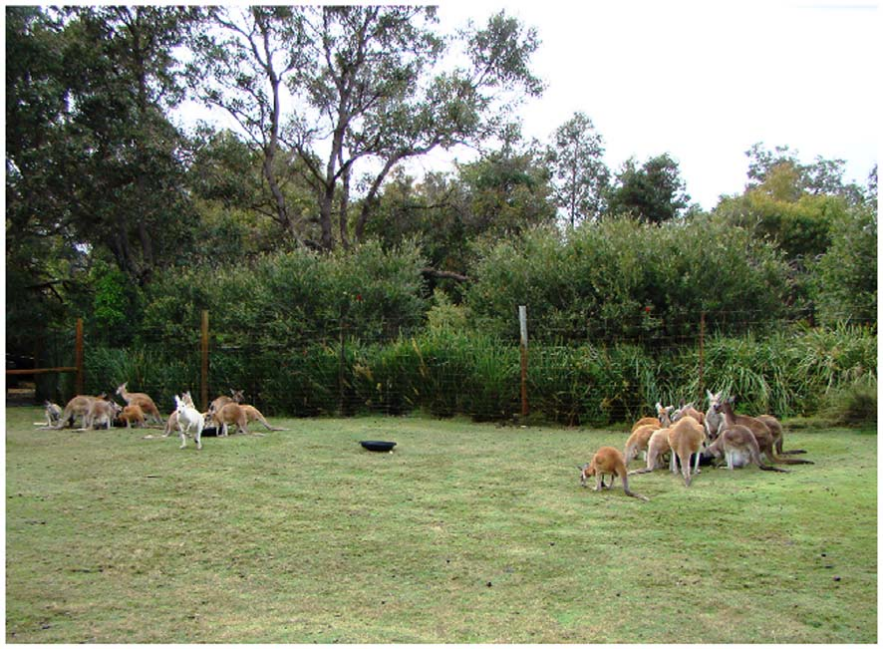
Cafeteria trials. Western grey (*Macropus fuliginosus*), red kangaroos (*Macropus rufous*) and agile wallabies (*Macropus agilis*) participating in cafeteria trials in Caversham Wildlife Park, Whiteman, WA. The treatment (dingo urine) is located beside the trough in the centre. Other treatments (feces, water) were rotated among troughs and position and differed according to day.

Cafeteria trials (Figure 3) were carried out from 27 September–6 October, 2007. The study area comprised a large grassy lawn on the eastern side of the property where 50–60 animals aggregated daily to interact and accept supplemental food from visitors. The property managers attracted the kangaroos to this area daily by offering the most palatable food one time/day (fresh breads at 6am). Two researchers were trained to follow the same feeding protocol as staff so that variations in animal participation may be attributed to treatments rather than undeclared variables. The kangaroos have followed this schedule for three years prior to our trials. Experiments were conducted before the park was opened for visitors. We employed feeding trials at four feeding stations [[14]; Figure 3] selected at arbitrary intervals. Animals had equal access to all stations.

Each station consisted of a single trough filled with 1 kg whole seeded-bread broken into 5 cm cubes. Four troughs were placed at approximately 4 m intervals along a linear transect. Despite our haphazard randomization of troughs to avoid handedness from influencing where animals select food, animals did not participate equally among the four troughs. Thus, we only included levels of food remaining in the trough beside treatment or tap-water control for comparative GUD measures (N = 10; 4 urine, 3 feces and 3 controls). Treatments consisted of 40 ml of dingo urine, 40 ml of tap water, or 20 mg of feces. Volumes/mass were chosen to represent a ‘typical’ void (Barry Oakman, Australian Dingo Conservation Association, personal communication) and placed next to one of the stations in a Petri dish.

Animals were observed by two observers, 10 m away (a distance that did not interfere with the behavior of these habituated subjects), and trials were terminated after 30 min. Remaining food at each trough was collected, and weighed to the nearest g to quantify the GUD. In addition to GUD, we recorded two behavioral measures that might indicate fear: flight from the feeding station, and foot stomps—an alarm signal that may function to warn conspecifics about imminent danger [47]; [48] or to confuse predators in pursuit [49]. We also used two measures to quantify altered palatability. We previously observed that kangaroos would turn away from an odor rather than leaning over the treatment to feed. Thus, we noted instances of feeding over the treatment trough, and refer to this behavior as ‘encroachment’.

We were unable to identify individual animals; therefore, we counted the number of animals participating in the trial area at the start of each trial as a crude measure of whether habituation was occurring over time.

### Statistics

We recorded four response variables to quantify approach and avoidance responses ([50]; Table 4): the frequency of flight, the number of foot stomps, number of encroaches directly over the treatment and z scores for the amount of biomass remaining (GUD). Kangaroo participation, defined as number of animals present in the trial area at the commencement of each trial, was recorded as a covariate for all measures. We fitted a MANOVA model in SPSS version 11.5 (SPSS Inc., Chicago, IL, U.S.A.) to all response variables. Tukey's HSD post hoc analysis was applied to within- subject treatments (e.g., urine, feces, control). A linear regression was fitted to assess whether elapsed days explained variation in participation rate. We calculated *d*-scores [51] to identify effect size of comparisons between treatments. All tests were two-tailed, we set our α = 0.05. Means are given ±SEM.
